# Lung Transplantation for Primary Ciliary Dyskinesia and Kartagener Syndrome: A Multicenter Study

**DOI:** 10.3389/ti.2023.10819

**Published:** 2023-02-14

**Authors:** Matteo Marro, Miguel M. Leiva-Juárez, Frank D’Ovidio, Justin Chan, Dirk Van Raemdonck, Laurens Joseph Ceulemans, Paula Moreno, Antonio Alvarez Kindelan, Thorsten Krueger, Angela Koutsokera, Jonas Peter Ehrsam, Ilhan Inci, Alkin Yazicioglu, Erdal Yekeler, Massimo Boffini, Geoffrey Brioude, Pascal Alexandre Thomas, Nikolaus Pizanis, Clemens Aigner, Marco Schiavon, Federico Rea, Marco Anile, Federico Venuta, Shaf Keshavjee

**Affiliations:** ^1^ Division of Cardiac Surgery, University of Turin, Turin, Italy; ^2^ Division of Thoracic Surgery, Columbia University Medical Center, New York, NY, United States; ^3^ Toronto Lung Transplant Program, University Health Network, Toronto, ON, Canada; ^4^ Department of Thoracic Surgery, University Hospitals Leuven, Leuven, Belgium; ^5^ Thoracic Surgery and Lung Transplantation Unit, University Hospital Reina Sofia, Cordoba, Spain; ^6^ Division of Thoracic Surgery, Lausanne University Hospital, Lausanne, Switzerland; ^7^ Department of Thoracic Surgery, University Hospital Zurich, Zurich, Switzerland; ^8^ Department of Thoracic Surgery, University of Health Sciences, Ankara, Türkiye; ^9^ Division of Thoracic Surgery, University of Marseilles, Marseille, France; ^10^ Department of Thoracic Surgery, University Hospital Essen, Essen, Germany; ^11^ Department of Cardio-Thoracic Surgery, Padua University Hospital, Padua, Italy; ^12^ Division of Thoracic Surgery, Policlinico Umberto I, Sapienza University, Rome, Italy

**Keywords:** outcomes, lung transplant, chronic lung allograft dysfunction, primary graft dysfunction, rare disease

## Abstract

Primary ciliary dyskinesia, with or without situs abnormalities, is a rare lung disease that can lead to an irreversible lung damage that may progress to respiratory failure. Lung transplant can be considered in end-stage disease. This study describes the outcomes of the largest lung transplant population for PCD and for PCD with situs abnormalities, also identified as Kartagener’s syndrome. Retrospectively collected data of 36 patients who underwent lung transplantation for PCD from 1995 to 2020 with or without SA as part of the European Society of Thoracic Surgeons Lung Transplantation Working Group on rare diseases. Primary outcomes of interest included survival and freedom from chronic lung allograft dysfunction. Secondary outcomes included primary graft dysfunction within 72 h and the rate of rejection ≥A2 within the first year. Among PCD recipients with and without SA, the mean overall and CLAD-free survival were 5.9 and 5.2 years with no significant differences between groups in terms of time to CLAD (HR: 0.92, 95% CI: 0.27–3.14, *p* = 0.894) or mortality (HR: 0.45, 95% CI: 0.14–1.43, *p* = 0.178). Postoperative rates of PGD were comparable between groups; rejection grades ≥A2 on first biopsy or within the first year was more common in patients with SA. This study provides a valuable insight on international practices of lung transplantation in patients with PCD. Lung transplantation is an acceptable treatment option in this population.

## Graphical Abstract

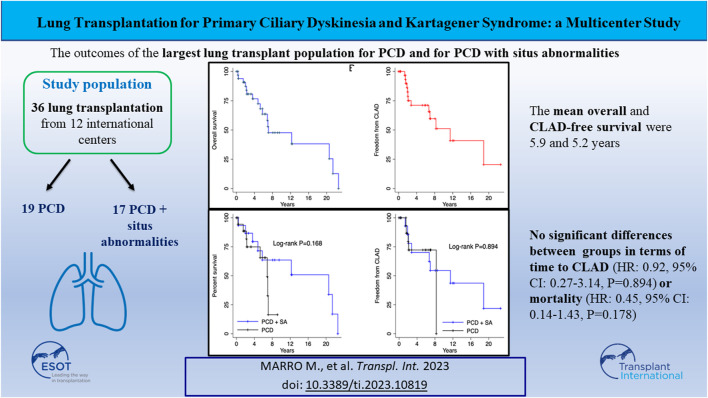

## Introduction

Primary ciliary dyskinesia (PCD) is an autosomal recessive disorder characterized by immotile, dysmotile, or absent cilia in the surface of cells of the airway, the reproductive system, and other tissues (1). The defect in ciliary motion leads to anomalous mucociliary clearance, resulting clinically in recurrent or persistent sinorespiratory infections and infertility. The prevalence of PCD is of 1 in 15,000–20,000 individuals. Given that its symptoms overlap with other respiratory diseases, PCD it is believed to be often under diagnosed or misdiagnosed (2,3).

Since normal ciliary function is necessary to control the cardiac laterality during embryologic development, a spectrum of organ laterality defects occur with PCD including situs inversus totalis and situs ambiguus. The triad of situs inversus, chronic sinusitis, and bronchiectasis has historically been referred to as Kartagener syndrome (KS), a subgroup today identified as PCD with situs abnormalities (4,5).

Less than 50% of PCD patients present with situs inversus totalis as per a complete transposition of the thoracic and abdominal viscera, whereas situs ambiguus with a partial transposition of thoracic and or abdominal viscera occurs in at least 12% of PCD. This condition—characterized by an arrangement of internal organs somewhere between situs solitus and situs inversus, can be associated with mild (cardiac septal defects) to severe (heterotaxy) congenital heart disease. In patients with PCD, further imaging studies such as abdominal ultrasound, CT scan, or echocardiogram are pivotal to detect subtle laterality defects (e.g., intestinal malrotation, interrupted inferior vena cava, or polysplenia). PCD should be considered in patients with persistent oto-sino-pulmonary symptoms and any organ laterality or cardiac defect (6–8).

PCD leads to severely impaired mucociliary clearance with recurrent respiratory tract infections and bronchiectasis, and otitis media with hearing loss. Productive cough, shortness of breath, chronic rhinitis and pansinusitis are typical presenting symptoms afflicting young patients during their childhood (9,10).

Patients with PCD are treated with chronic suppressive antibiotics, bronchodilators and inhaled hyperosmolar agents combined with chest physiotherapy to promote airway clearance, vaccination to prevent new upper and lower respiratory infections and on-demand antibiotics for acute exacerbations (9). Lung transplantation is an option for patients with end-stage PCD resulting in respiratory insufficiency (9,11). According to the thirty-sixth registry report by the International Society for Heart and Lung Transplantation (ISHLT) (12), non-cystic-fibrosis bronchiectasis represent 0.4% of all single-lung transplants and 3.8% of all double-lung transplants since 1995. However, lung transplantation in patients with PCD with or without situs abnormalities has been described in only a few case reports (9,13–15) and series (11,16). Therefore, we sought to investigate the outcomes of patients receiving lung transplantation for PCD with or without situs abnormalities across an international multicenter effort promoted by the European Society of Thoracic Surgeons (ESTS) Lung Transplantation Working Group on Rare Diseases.

## Materials and Methods

### Patient Population and Study Design

This retrospective multicenter study was conducted by the ESTS Lung Transplantation Working Group and it was established bridging off the larger study on rare indications for lung transplantation; the study was open to non-European centers (United States, Canada, and Turkey). A total of 36 lung transplant recipients for PCD and KS from 1995–2020 were included in the study.

### Data Source

Data for this study was retrospectively recorded from the participating center archives. Patient data was anonymized and collected in a dedicated database after a data transfer agreement was signed, when required. Variables collected included patient demographic characteristics, diagnoses, information on type of transplantation, induction immunosuppressive therapy and follow-up.

Data was summarized and analyzed in the primary study center (Columbia University Medical Center). PCD was defined as the presence of a genetic mutation related to ciliary motility or a structural defect in electron microscopy after cystic fibrosis was ruled out. KS was defined as the confirmed diagnosis of PCD plus any sign of situs abnormalities. Records were eligible for inclusion if the patient received his or her first lung transplantation for PCD with or without a situs abnormality. Diagnoses were established before transplantation and were attributed independently by each center. No time limits were established for the patient enrollment.

### Outcomes and Study Definitions

Primary outcomes of interest included survival and freedom from chronic lung allograft dysfunction (CLAD). CLAD was defined in accordance to the ISHLT consensus statement as a persistent (lasting more than 3 months) and irreversible decline in the forced expiratory volume after 1 s (FEV_1_) ≥ 20% from the post-transplant baseline. This was identified the average of the two maximal post-transplant FEV_1_ values monitored at least 3 weeks apart, with absent clinical confounders (12). This definition has been retrospectively adopted for all patients. Secondary outcomes of interest included primary graft dysfunction (PGD—with the definition and grading by the report of the ISHLT in 2016) (17) within the first 24, 48, and 72 h and the rate of rejection ≥A2 within the first year and first biopsy, along with predicted FEV_1_ volumes at different timepoints. We decided to consider as significant only rejections equal or more than mild; symptoms may be more frequent in patients with grade A2 or higher compared with those with grade A0 or A1 (18).

The protocol was created in adherence to the Institutional Review Board of the Columbia University Medical Center (IRB: AAAT0932).

### Statistical Analysis

Continuous and categorical variables were compared for measures of central tendency and rates. Differences in continuous and categorical variables were compared using a Mann-Whitney (or a Student’s t-test for normal distributions) and *χ* ^2^ test, respectively. Normality was inferred from both visual analysis of distributions and using a Shapiro-Wilk test. Time-to-event data was displayed using Kaplan-Meier plots and tested using a log rank test. A *p* < 0.05 was considered statistically significant. All statistical analyses were performed using Stata Version 14.0 (StataCorp, College Station, TX, United States).

## Results

### Patient Demographics

Eight European and three non-European lung transplantation centers participated in the study (Supplementary Table S1). Clinical records of 36 patients with end-stage severe respiratory failure were extracted and collected and baseline demographic and clinical data were analyzed. Of these, 52.8% (*n* = 19) had PCD and 47.2% (*n* = 17) had PCD + SA. Donor characteristics were similar between patients with PCD compared with those with situs abnormalities, with exception of a higher rate of donors with smoking history in the PCD group (3% vs. 1%, *p* = 0.031) and overall higher donor and recipient height (Table 1). No relevant comorbidities were reported.

**TABLE 1:** Donor and recipient demographics.

		Overall (*n* = 36)		PCD (*n* = 19)		PCD + SA (*n* = 17)		*p*-value
		Median	[IQR]	Median	[IQR]	Median	[IQR]	
Donor demographics								
Donor age (years)		33.5	[25–49.5]	39	[25–62]	28	[20–44]	0.061
Donor height (cm)		172	[165–177.9]	169	[163–172.7]	174.5	[170–180.3]	0.02
Donor weight (kg)		75	[65.5–81]	77.8	[68.4–87.5]	73.56	[65–80]	0.421
P/F at 100%		479	[393–533]	478	[393–531]	490	[393–540]	0.597
P/F at 40%		180	[156–204]	170	[156–202]	197	[165–204]	0.516
		***n***	**%**	***n***	**%**	***n***	**%**	***p*-value**
Donor gender								
Male		20	55.6	11	57.9	9	52.9	0.765
Female		16	44.4	8	42.1	8	52.9	
Donor cause of death								
Anoxia		1	2.8	0	0.0	1	5.9	0.232
PE		1	2.8	1	5.3	0	0.0	
Trauma		8	22.2	3	15.8	5	29.4	
ICH/CVA		21	58.3	11	57.9	10	58.8	
Cardiac failure		1	2.8	1	5.3	0	0.0	
Donor smoker		4	11.1	3	15.79	1	5.9	0.031
		**Median**	**[IQR]**	**Median**	**[IQR]**	**Median**	**[IQR]**	***p*-value**
Recipient demographics								
Age at listing (years)		42.5	[32.5–53.5]	39	[32–48]	48	[34–55]	0.715
Recipient age at LTx (years)		43.1	[34.5–56]	39.9	[35–50]	48	[34–56]	0.812
Recipient height (cm)		169	[157.5–173]	163	[153–170.5]	172	[169–175]	0.009
Recipient weight (kg)		58.2	[53–65]	55	[48.2–64]	62.15	[55–71]	0.051
Waitlist time (days)		284	[77–558]	366.5	[131–626]	240	[77–415]	0.531
Pre-op systolic PAP (mm Hg)		38	[30–50]	38	[35.5–48]	36.5	[24–50]	0.552
Pre-op mean PAP (mm Hg)		24	[20–33]	23	[20–28]	29	[19–38]	0.274
Pre-op Wedge pressure (mm Hg)		10	[9–14]	9.5	[7.5–14]	13	[10–14]	0.21
Pre-op CO (L/min)		4.4	[3.2–5.5]	4.4	[3.4–5.5]	3.9	[2.9–5.4]	0.734
		***n***	**%**	***n***	**%**	***n***	**%**	***p*-value**
Recipient gender								
Male		17	47.2	7	36.8	10	58.8	0.187
Female		19	52.8	12	63.2	7	41.2	
Preop mechanical ventilation (days)		2	5.6	0	0.0	2	11.8	0.161
Preop ECMO (days)		1	2.8	0	0.0	1	5.9	0.306
Urgent priority		3	8.3	0	0.0	3	17.6	0.056

PCD, primary ciliary dyskinesia, SA, situs abnormalities, PAP, pulmonary arterial pressure, CO, cardiac output, PE, pulmonary embolism, ICH, intracranial hemorrhage, CVA, cerebrovascular accident, ECMO, extracorporeal membrane oxygenation, LTx, lung transplantation; P/F, ratio of artieral partial pressure of oxygen to inspired oxygen concentration.

Four patients, 2 (10.5%) in the PCD group and 2 (11.8%) in the PCD + SA group, presented chronic colonization by *Pseudomonas aerouginosa* and *Serratia marcescens*. All of them were treated pre-surgery by chronic antibiotic therapy with azithromycin and transplanted with no ongoing infection.

None of our patient underwent lobectomy or segmentectomy prior to LTX. As suggested by Kouis et al. (19), prevalence of lung resection in PCD is often performed before PCD diagnosis and overall is more frequent in patients with delayed diagnosis. After lung resection, lobectomised patients have poorer and continuing decline of lung function despite lung resection.

All PCD patients with situs abnormalities had situs inversus totalis. The median age of listing was 42.5 years [IQR: 32.5–53.5] with a median waiting list time of 284 days [IQR: 77–558]. The age at time of surgery ranged from 15 to 66 years (median: 43.1, IQR: 34.5–56). Preoperative hemodynamic parameters, mechanical ventilation and extra corporeal membrane oxygenator (ECMO) use were similar between groups. Patients were followed for a median of 4.23 years (range: 0.25–22.6 years) and included transplants performed between 1999 and 2020.

### Perioperative Characteristics

The median cold ischemic time for the first and second lung were 281 [IQR: 181–375] and 417 [IQR: 308–499] min, respectively, and no differences were seen between subdiagnoses (Table 2). All the recipients transplanted with urgent priority were PCD + SA patients (0% vs. 17.6%). The majority of the patients received double lung transplant (88.9%) with a similar rate by each subdiagnosis. Two patients received single LTx due to the absence of pre-operative bacterial colonization and the center-managements strategies, while two patients underwent a heart-lung transplantation at a single institution because of the presence of severe congenital heart defect. No special procedure were required during surgeries. *Ex vivo* lung perfusion technique was performed in two cases, both in the PCD group. The rates of intraoperative and postoperative ECMO were similar among patients with and without a situs abnormality. Seventeen patients (47.2%) received induction immunosuppression, which was based on anti-interleukin-2 receptor (*n* = 13, 36.1%) or anti-thymocyte globulin (*n* = 4, 11.1%) antibodies. The individual type of maintenance immunosuppression also varied by center. The majority of patients were treated by a combination of tacrolimus, mycophenolate mofetil and corticosteroids (Table 2). No significant differences between maintenance immunosuppressive therapies were observed between patients with or without situs abnormalities.

**TABLE 2:** Perioperative characteristics.

		Overall (*n* = 36)		PCD (*n* = 19)		PCD + SA (*n* = 17)		*p*-value
		Median	[IQR]	Median	[IQR]	Median	[IQR]	
Cold ischemic time first lung (min)		281	[181–375]	271	[225–330]	291	[220.5–352]	0.812
Cold ischemic time second lung (min)		417	[308–499]	429	[335–460]	398.5	[332.5–498]	0.82
Time to extubation (hours)		36	[24–69]	36	[20–96]	33	[20–92]	0.817
		***n***	**%**	***n***	**%**	***n***	**%**	***p*-value**
Intraoperative CPB/ECMO		17	47.2	8	42.1	9	52.9	0.516
EVLP		2	5.6	2	10.5	0	0.0	0.169
Type of transplant								
Heart-lung		2	5.6	0	0.0	2	11.8	0.302
Double		32	88.9	18	94.7	14	82.4	
Single		2	5.6	1	5.3	1	5.9	
Postop ECMO								
VV		1	2.8	1	5.3	0	0.0	0.509
VA		3	8.3	1	5.3	2	11.8	
Induction immunosuppression^a^							
None		18	50.0	7	36.8	11	64.7	0.362
Anti-thymocyte globulin		4	11.1	3	15.8	1	5.9	
Anti IL-2r		13	36.1	8	42.1	5	29.4	
Maintenance immunosuppression							
Cyclo		3	8.3	1	5.3	2	11.8	0.415
Cyclo + AZA + CS		4	11.1	3	15.8	1	5.9	
Cyclo + MMF + CS		7	19.4	3	15.8	4	23.5	
Tacro + MMF		3	8.3	3	15.8	0	0.0	
Tacro + MMF + CS		14	38.9	6	31.6	8	47.1	
Tacro + Ever + CS		1	2.8	0	0.0	1	5.9	
Tacro + CS		3	8.3	2	10.5	1	5.9	

^a^ Data from a PCD patient is not available.

PCD, primary ciliary dyskinesia; SA, situs abnormalities; CPB, cardiopulmonary bypass; ECMO, extracorporeal membrane oxygenation; EVLP, *ex vivo* lung perfusion; VA, venoarterial; VV, venovenous; AZA, azathioprine; MMF, mycophenolate mophetil; CS, corticosteroids; Anti-IL-2r, anti-interleukin-2 receptor; Ever, Everolimus; Cyclo, cycloporine; Tacro, tacrolimus.

### Postoperative Outcomes

**FIGURE 1:**
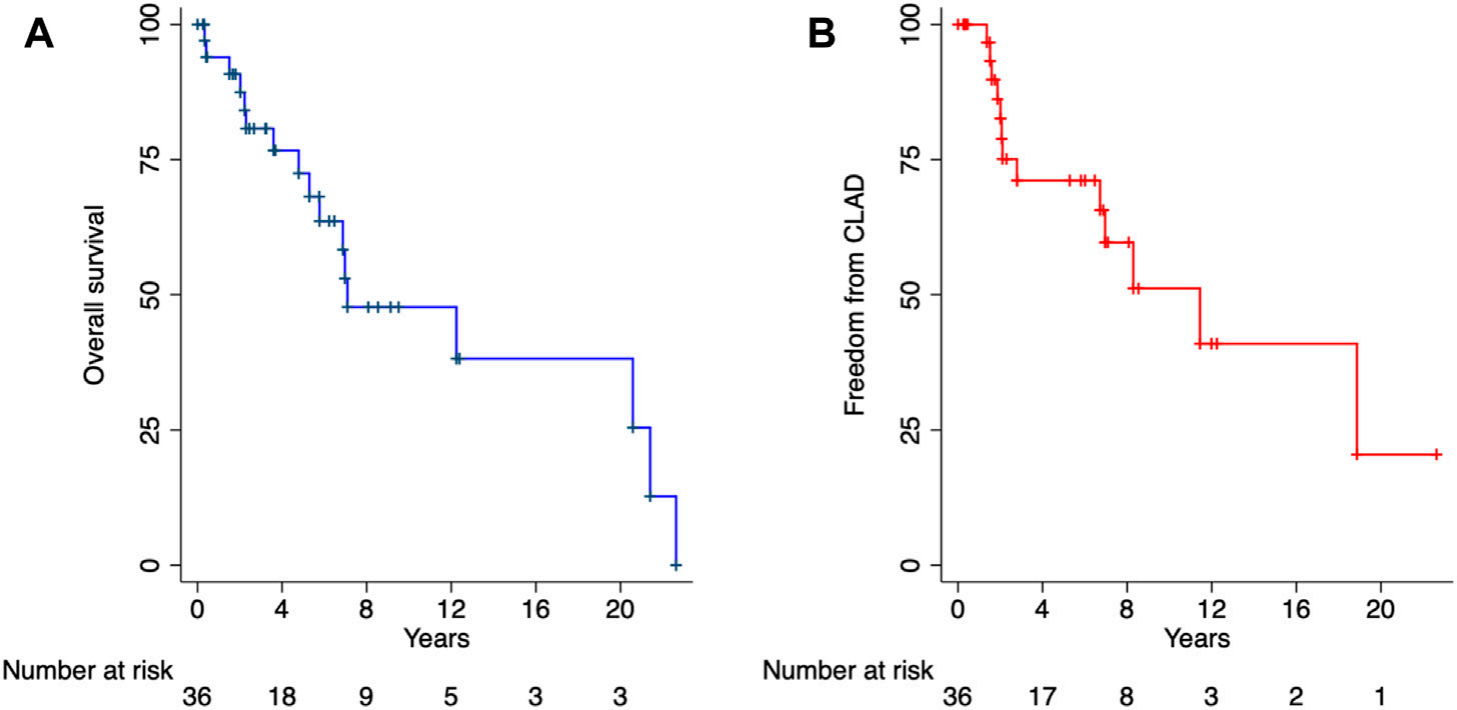
Overall survival **(A)** and freedom from chronic lung allograft dysfunction **(B)**. CLAD, chronic lung allograft dysfunction.

**FIGURE 2:**
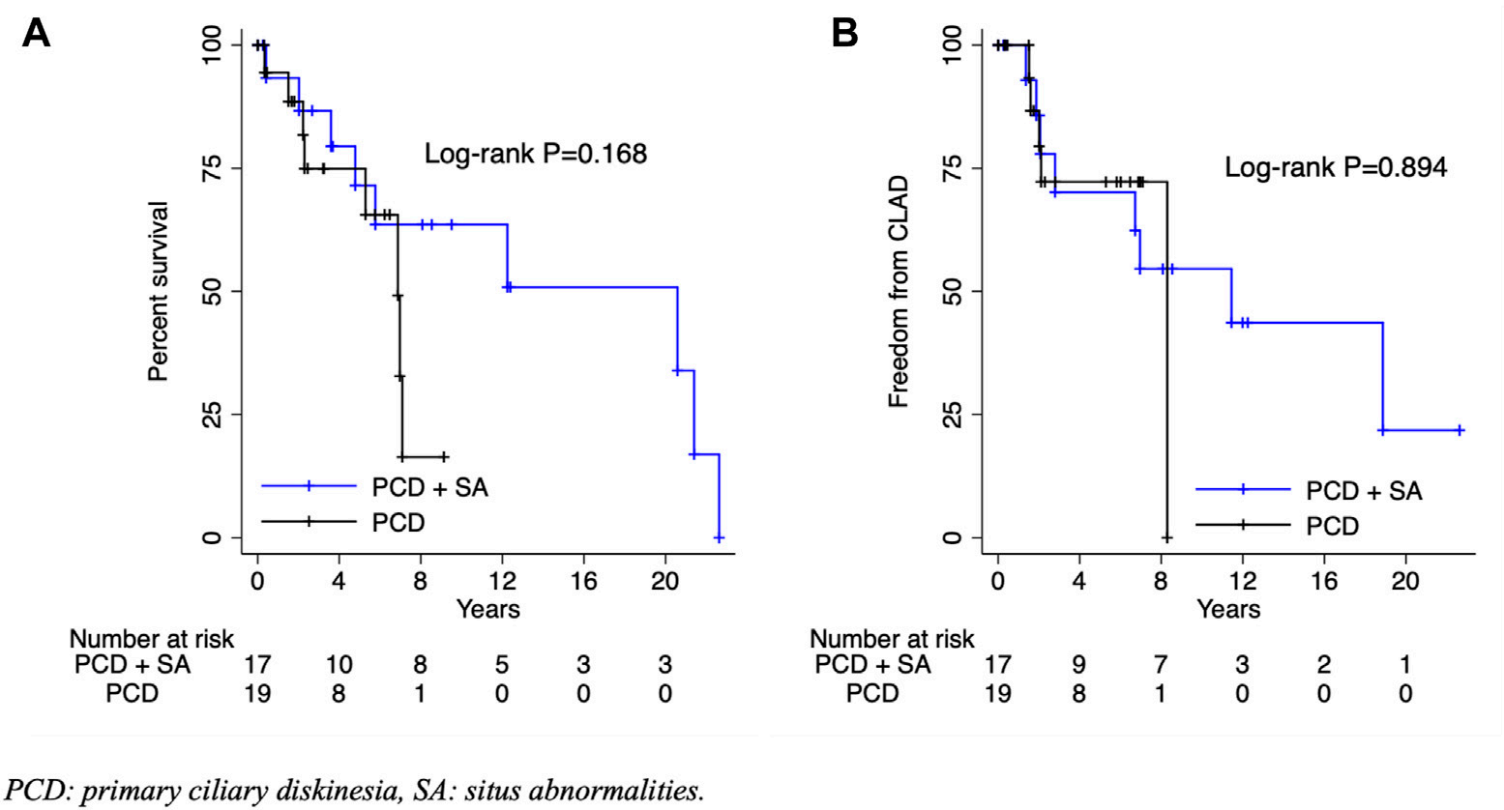
Overall survival **(A)** and freedom from chronic lung allograft dysfunction **(B)** by presence of situs abnormalities. PCD, primary ciliary diskinesia; SA, situs abnormalities; CLAD, chronic lung allograft dysfunction.

The primary outcomes of interest were mortality and freedom from CLAD. Among patients with PCD with or without SA, the mean overall and CLAD-free survival were 5.9 and 5.2 years, respectively (*p* = 0.894) (Figure 1). There were no significant differences in the time to CLAD (PCD + SA; HR: 0.92, 95% CI: 0.27–3.14, *p* = 0.894) or mortality (PCD + SA; HR: 0.45, 95% CI: 0.14–1.43, *p* = 0.178) between PCD and KS groups (Figure 2). The median ICU and total length of stay after transplantation were 7 [IQR: 4–14] and 31.5 [IQR: 20–45] days, respectively. Patients with KS had longer ICU stays (5 vs. 12 days, *p* = 0.029) and a trend towards a longer total length of stay (26 vs. 41 days, *p* = 0.114) (Table 3). Postoperative rates of PGD within the first 72 h were comparable between groups; rejection grades ≥A2 on first biopsy or within the first year was more common in patients with KS, although no statistical significant difference was noted.

**TABLE 3:** Postoperative outcomes.

		Overall (*n* = 36)		PCD (*n* = 19)		PCD + SA (*n* = 17)		*p*-value
		Median	[IQR]	Median	[IQR]	Median	[IQR]	
ICU length of stay (days)		7	[4–14]	5	[2–11]	12	[7–26]	0.029
Total length of stay (days)		31.5	[20–45]	26	[20–42]	41	[19–55]	0.114
Percent predicted FEV1								
3 months		77	[61–86]	73.5	[61–85]	80	[64–87]	0.734
6 months		80	[63–92]	83	[57–92]	77	[68–92]	0.550
1 year		80	[57–90]	80	[57–90]	91	[62.3–90]	0.864
Latest		70	[41–88]	74	[46–97]	56	[36–76]	0.233
		***n***	**%**	***n***	**%**	***n***	**%**	***p*-value**
PGD at 24 h^a^								
PGD 0		17	47.2	7	36.8	10	58.8	0.407
PGD 1		11	30.6	8	42.1	3	17.6	
PGD 2		4	11.1	2	10.5	2	11.8	
PGD 3		3	8.3	2	10.5	1	5.9	
PGD at 48 h^a^								
PGD 0		17	47.2	6	31.6	11	64.7	0.067
PGD 1		14	38.9	11	57.9	3	17.6	
PGD 2		3	8.3	2	10.5	1	5.9	
PGD 3		1	2.8	0	0.0	1	5.9	
PGD at 72 h^a^								
PGD 0		18	50.0	7	36.8	11	64.7	0.371
PGD 1		11	30.6	8	42.1	3	17.6	
PGD 2		3	8.3	2	10.5	1	5.9	
PGD 3		2	5.6	1	5.3	1	5.9	
Rejection ≥A2 on first biopsy	9	25.0	3	15.8	6	35.3	0.177
Rejection ≥A2 within first year		6	16.7	1	5.3	5	29.4	0.052
CLAD		13	36.1	5	26.3	8	47.1	0.196

^a^ Data from a PCD + SA patient is not available.

PCD, primary ciliary dyskinesia; SA, situs abnormalities; ICU, intensive care unit; FEV1, forced expiratory volume at 1 s; PGD, primary graft dysfunction; CLAD, chronic lung allograft dysfunction.

All patients with onset of CLAD were treated with azitromicin at the beginning of respiratory function decline. Until today, none of them underwent a re-transplant.

## Discussion

This manuscript reports the findings of a multicenter retrospective study with the largest lung transplant population for PCD with and without situs abnormalities to date. The demographic analysis between the two groups does not highlight specific differences except for the recipient height, and the cold ischemic time for the first and second lung is longer for the PCD + SA group but with no statistically significant differences. This finding could be explained by the inverted anatomy of the PCD + SA patient resulting in increased surgical difficulty and time required by the surgeon for the transplant. No other differences have been found between the two groups in terms of peri-operative characteristics. Considering the number of the involved centers, some minor surgical technique differences are present: the bronchial anastomosis performed by a continues or interrupted suture, differences of type of sutures and/or size, and different technical approach for double lung transplant (double antero-lateral thoracotomies or Clam Shell approach).

The majority of the patients received tacrolimus, mycophenolate mofetil and corticosteroids as immunosuppressive therapy. Recipients with situs abnormalities had a longer ICU stay and total length of in-hospital stay.

Minor difference were noted in the use of extra corporal support in PCD with situs abnormalities recipients which may justify the postoperative outcome with longer ICU stays.

This multicenter report shows that approximately 70% of the total population is alive 5 years after transplantation with no difference between the two groups. In their analysis from the UNOS registry, Hayes reported a 5-year survival of 52% for PCD and 65% for KS (11). Analysis of the ISHLT registry had previously reported survival rates around 80% at 1 year and 54% at 5 years for the 63,410 adults who underwent primary lung transplantation between January 1992 and June 2017 (12). The better 5-year survival rate of our population compared with the population from the 38th ISHLT Registry could be explained by the younger age of our recipients (43.1 years vs. 54.8 years, respectively) (20). Indeed, PCD and KS patients, due to the natural course of their disease, are listed and transplanted at an earlier age compared to the global LTx population.

We report freedom from CLAD of approximately 50% at 10 years, improved from previous reports by Sato (less than 50% at 8 years from transplant) (21) and from the 38th ISHLT Registry for the overall population (57% at 5 years for the era 1996–2001, 49% for the era 2002–2007 and 47% for the era 2008–2013) (20). Moreover, this registry data show a better 5-year freedom from CLAD for recipients with cystic fibrosis compared with chronic obstructive pulmonary disease and idiopatic pulmonary fibrosis diagnosis at transplant. The maintenance of immunosuppression in terms of blood levels, type of therapy, prevention of cytomegalovirus replication might affect and delay the CLAD onset. Additionally, there were no significant differences in the time to CLAD or mortality in PCD with or without situs abnormalities.

In our cohort CLAD was more common in patients treated with cyclosporine than with tacrolimus (64.3% vs. 19%, but there was not statistically significant differences). This is in accordance with prior studies that demonstrated an improvement in incidence of acute rejection and long-term outcomes including a reduced risk for CLAD in patients treated with tacrolimus (22).

Since PCD + SA can be associated with spinal scoliosis and substantial distortion of the intrathoracic space, size matching is particularly important in these patients undergoing lung transplantation. A detailed preoperative study with computed tomography 3D reconstruction to better evaluate the anatomy in these patients and to precisely plan the surgery should be considered by the transplant team (23).

The main surgical transplant pitfalls in patients with situs inversus are the complete reversal of the anatomic position of both cardiac chambers and main vessels and the inverse direction and position of both lungs and main stem bronchi, leading to an anatomical mismatch between the recipient and the donor (16). However, the atrial and pulmonary anastomoses can be performed without difficulties due to the midline position of the left atrium and two pulmonary arteries.

To avoid and prevent possible surgical mismatch it is important to obtain a long atrial cuff from the donor and retain maximal length of both the donor and recipient pulmonary arteries.

In patients with situs abnormalities, bronchoscopic findings are consistent with anatomic reversal of the morphology of right and left airways, which include a long right main and a short left main bronchus with early take off of the left upper lobe. Furthermore, the recipient left pulmonary artery can be located anterior to the bronchus while the donor left pulmonary artery may be located superior to the bronchus. Several groups have described methods to address this mismatch. Gauthier described a generous vascular mobilization of the recipient left pulmonary artery from a prebronchial position to an epibronchial position, tailoring the arteriotomies to facilitate an end-to-end vascular anastomosis (24). Another approach described by Mentzer facilitated the anastomosis of the left pulmonary artery by a termino-lateral anastomosis between the donor left pulmonary artery and the recipient origin of the truncus anterior artery, preventing possible distortion and obstruction of the pulmonary vessel (25). Furthermore De Castro suggested, to prevent a left pulmonary artery kinking, to leave a shorter pulmonary artery stump during the graft back table preparation (26).

Moreover, previous groups have emphasized the potential anatomical size mismatch between the donor’s right lower lobe and the recipient’s dextrocardia (15,25). Although in our cohort lung volume reduction surgery was not necessary, Macchiarini described right lower lobectomy during lung transplantation because of excessive volume of the right donor lung (16).

In terms of pre-operative evaluation and preparation for anesthesia, the use of a conventional right-sided double-lumen tube placed in the anatomic right main bronchus rather than a standard left sided double-lumen tube might overcome the inverted anatomy of airways, thus facilitating excision of the anatomic left lung with division of the short recipient left main bronchus (24).

Concerning the postoperative persistent lack of mucociliary clearance in the upper and central airways, all centers involved in this study used to perform pre-transplant bronchoscopy at the time of surgery. In the post-operative course, chest physiotherapy with deep breathing exercises, postural drainage combined with percussion, vibration and forced expirations, positive expiratory pressure (PEP) valves are routinely used to favor the mucous clearance.

None of the centers involved adopted prophylaptic tracheostomy to facilitate upper airway management and only two patients underwent surgical tracheostomy due to a prolonged respiratory weaning.

### Limitations

There are several limitations related to the design and population of this study. First, this study is limited by the effects of small sample size typical of rare diseases. As a consequence, no reliable analysis could be performed to predict long-term outcomes and the lack of significant differences between groups could be due to a lack of power. Additionally, information on patient selection and listing process was not available. The approach to donors has changed over time, not in terms of selection, but rather due to the ongoing improvement of ICU management, of the arrangement of organ donation, and the introduction of EVLP technique have allowed for a better quality pool of grafts. Variation in individual center selection criteria could potentially affect the overall outcomes for patients suffering from this condition. Moreover, given the low number of patients, the data was collected over a long period of time which could potentially insert time effect bias. This study, however, provides a valuable scope on international practices of lung transplantation in patients with PCD despite situs abnormalities.

### Conclusion

We have reported the largest multicenter study cohort of lung transplant in PCD patients with or without situs abnormalities. Our results confirm that, considering surgical pitfalls, lung transplantation is a feasible therapeutic option allowing long-term survival in patients with end-stage PCD with or without situs abnormalities.

## Data Availability

The datasets presented in this study can be found in online repositories. The names of the repository/repositories and accession number(s) can be found in the article/Supplementary Material.
