# Supplementary material for: Lung Transplantation for Primary Ciliary Dyskinesia and Kartagener Syndrome: A Multicenter Study
Source: Transpl Int. 2023 Feb 14;36:10819. doi: 10.3389/ti.2023.10819 (PMC9970992; doi:10.3389/ti.2023.10819)
Supplement: Supplementary file 1 [file Table1.docx]

| Centers | | Number of Cases | | |  |
| --- | --- | --- | --- | --- | --- |
|  | | Total | PCD | PCD + SA |  |
| Toronto General Hospital, University of Toronto | | 8 | 5 | 3 |  |
| Columbia University Medical Center | | 6 | 4 | 2 |  |
| University Hospitals Leuven | | 5 | 3 | 2 |  |
| Zurich University Hospital | | 3 | 1 | 2 |  |
| University Hospital Reina Sofia, Cordoba | | 3 | 0 | 3 |  |
| Centre Hospitalier Universitaire, Lausanne-Geneva | | 3 | 3 | 0 |  |
| University of Health Sciences Turkey, Ankara | | 2 | 0 | 2 |  |
| Turin University | | 2 | 1 | 1 |  |
| University Medicine Essen | | 1 | 0 | 1 |  |
| Marseille University | | 1 | 0 | 1 |  |
| Rome Sapienza University | | 1 | 1 | 0 |  |
| Padua University Hospital | | 1 | 1 | 0 |  |
| Total | | 36 | 19 | 17 |  |
|  | *PCD: primary ciliary dyskinesia; SA: Situs Abnormalities.* | | | | |

**Supplementary Table 1. Participating centers and patient numbers**
